# Detecting and Characterizing A-To-I microRNA Editing in Cancer

**DOI:** 10.3390/cancers13071699

**Published:** 2021-04-03

**Authors:** Gioacchino P. Marceca, Luisa Tomasello, Rosario Distefano, Mario Acunzo, Carlo M. Croce, Giovanni Nigita

**Affiliations:** 1 Department of Clinical and Experimental Medicine, University of Catania, 95125 Catania, Italy; 2 Department of Cancer Biology and Genetics and Comprehensive Cancer Center, The Ohio State University, Columbus, OH 43210, USA; luisa.tomasello@osumc.edu (L.T.); rosario.distefano@osumc.edu (R.D.); carlo.croce@osumc.edu (C.M.C.); 3 Division of Pulmonary Diseases and Critical Care Medicine, Virginia Commonwealth University, Richmond, VA 23298, USA; mario.acunzo@vcuhealth.org

**Keywords:** A-to-I RNA editing, ADAR, microRNAs, microRNA targeting, detection, quantification, functional characterization

## Abstract

**Simple Summary:**

Adenosine to inosine (A-to-I) editing is a type of RNA editing where individual adenosines are enzymatically converted into inosines. A-to-I RNA editing plays an important role in cancer biology. Several studies have demonstrated that A-to-I editing of microRNAs (miRNAs) very often affect miRNA function as oncosuppressors or oncogenes, hence showing clinical relevance. Hence, A-to-I miRNA editing has been suggested as a potential diagnostic and prognostic tool in the monitoring of cancer patients. Nevertheless, the process of identifying and characterizing miRNA editing events in tumor samples still presents several challenges. In this review, we outline molecular aspects linked to miRNA A-to-I editing and retrace methods and approaches dedicated to detection of editing sites and functional characterization of edited miRNAs in cancer.

**Abstract:**

Adenosine to inosine (A-to-I) editing consists of an RNA modification where single adenosines along the RNA sequence are converted into inosines. Such a biochemical transformation is catalyzed by enzymes belonging to the family of adenosine deaminases acting on RNA (ADARs) and occurs either co- or post-transcriptionally. The employment of powerful, high-throughput detection methods has recently revealed that A-to-I editing widely occurs in non-coding RNAs, including microRNAs (miRNAs). MiRNAs are a class of small regulatory non-coding RNAs (ncRNAs) acting as translation inhibitors, known to exert relevant roles in controlling cell cycle, proliferation, and cancer development. Indeed, a growing number of recent researches have evidenced the importance of miRNA editing in cancer biology by exploiting various detection and validation methods. Herein, we briefly overview early and currently available A-to-I miRNA editing detection and validation methods and discuss the significance of A-to-I miRNA editing in human cancer.

## 1. Introduction

The presence of various RNA modifications has long been recognized. However, only recently, the study of their occurrence and function has received attention [1]. To date, it is known that RNA modifications are more prevalent and chemically diverse than those occurring in DNA, with over 70 ascertained post-transcriptional RNA biochemical alterations in eukaryotes [2]. Among these, adenosine to inosine (A-to-I) RNA editing is one of the best-characterized. It consists of the irreversible conversion of adenosine to inosine and is catalyzed by adenosine deaminase acting on RNA (ADAR) enzymes, specifically ADAR1 and ADAR2.

ADARs are evolutionarily conserved [3] and finely regulated by specific transcriptional factors throughout the various phases of development [4]. Over the last years, the expression pattern and functions of ADARs have been better defined, evidencing a role in tumor-related processes. ADAR1, which comprises two distinct isoforms, i.e., p110 and p150, is ubiquitously expressed [5]. It is involved in organogenesis, hematopoiesis (both isoforms), and suppression of immune response mediated by double-stranded RNA (dsRNA)-activated immunoproteins like the RIG-I-like receptor MDA5 and the interferon-induced PKR (p150 only) [6,7,8]. Indeed, recent research has shown that suppression of the cytosolic innate immune system might be the most prominent function of the ADAR1-mediated editing [7,8,9]. Data analyses from various tumor cell lines and cancer patients let emerge that ADAR1 RNA editing is essential for the survival of a subset of cancer cell lines. In fact, the editing activity of this enzyme was shown to prevent the recognition of edited dsRNAs by MDA5 and PKR, which are, respectively, a trigger of the type I interferon signaling and a growth inhibitor. Accordingly, ADAR1 depletion rescued the MDA5 and PKR-mediated lethality [10,11] and sensitized tumors to immunotherapy overcoming resistance to checkpoint blockade [12]. Hence, ADAR1 has been proposed as a potential therapeutic target in a subset of cancers. ADAR2 is mainly expressed in the nervous system [5] and contributes to modulating proliferation of neuronal and glial cells, ion channel activity and release of neurotransmitters, with repercussions on nervous system development, behavior, and the sleep–waking cycle [13,14,15]. Several studies have demonstrated that ADAR2 acts as a tumor-suppressor in various brain cancers. ADAR2 editing was significantly impaired in the context of glioblastomas and astrocytoma, favoring tumor progression. In contrast, the restoration of ADAR2 editing resulted in the inhibition of tumor cell growth and proliferation both in vitro and in vivo [16,17,18,19]. In this regard, an elucidated mechanism concerns the ADAR2-mediated control of the dual-specificity protein phosphatase *CDC14B* expression. ADAR2 is constitutively expressed in normal white matter, and its catalytic activity induces the expression of the *CDC14B* gene. Meanwhile, ADAR2 itself stabilizes the *CDC14B* transcript by editing it [19]. CDC14B is involved in the exit of cell mitosis and initiation of DNA replication and acts on the Skp2/p21/p27 pathway, which has a pivotal role in the G1/S checkpoint. The downregulation of ADAR2 in glioblastomas and astrocytoma hence causes a significant under-expression of CDC14B, with a consequent unbalance in cell cycle progression [19]. Altogether, these facts have increasingly prompted the research on A-to-I RNA editing in oncology.

Initially, the research on ADARs enzymatic activity focused on editing sites falling into protein-coding regions of mRNAs [20]. However, the employment of ever-powerful sequencing technologies and innovative bioinformatic pipelines evidenced that the vast majority of A-to-I RNA editing falls into non-coding regions of the human genome. In particular, RNA duplexes derived from inverted Alu repetitive elements represent the main targets of ADAR enzymatic activity, followed by RNA duplexes from non-Alu repetitive elements and non-repetitive elements, respectively [21,22,23].

To date, an overwhelming number of A-to-I editing sites in non-coding RNA (ncRNA) classes have been reported, though functional roles for most of them have remained mostly elusive [24]. A-to-I editing in microRNAs (miRNAs) represents one of the few exceptions, as its functional roles have been demonstrated, especially in the context of cancer development and progression. Hereafter, we elucidate the role of A-to-I miRNA editing in human cancers and briefly discuss various approaches to detect and validate such a modification in miRNAs.

## 2. MiRNA Biogenesis and Function in Cancer

**Figure 1:**
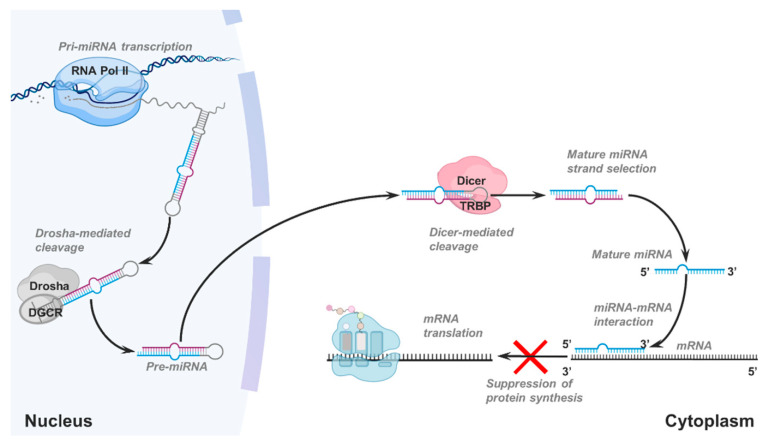
Illustration of canonical microRNA (miRNA) biogenesis. Once primary miRNA is transcribed, they are processed by the Drosha-DGCR8 complex and then exported to the cytoplasm. Here, pre-mRNA molecules are further processed by the Dicer-TRBP complex, usually generating two paired, partially complementary, mature miRNAs. One of the mature miRNA strands will be maintained while the other will be degraded. The selected miRNAs function as inhibitors of mRNA translation, usually by base-pairing of their seed region with complementary regions within the 3’ untranslated regions of the mRNAs.

MiRNAs are single-stranded ncRNA molecules composed of ~21 nucleotides, acting as translation inhibitors. MiRNAs are initially transcribed as independent genes or excised from their hosting genes’ introns [25,26]. Mature miRNA sequences are located in short stem-loop regions, which are cleaved from pri-miRNAs by the RNase III Drosha in complex with its cofactor DGCR8. Such processing gives rise to ~70 nucleotide long RNA hairpin molecules termed miRNAs precursors (pre-miRNAs). Pre-miRNAs are exported to the cytoplasm, where they are further processed by the RNase III Dicer in complex with its cofactor TRBP. The Dicer cleavage usually generates two mature miRNAs (miRNA-5p and miRNA-3p), but only one is maintained as a functional miRNA [27] (Figure 1).

The basic requirement for miRNAs to exert their repressive role in gene expression is a thermodynamically stable base pairing between nucleotides 2–8 at the 5’ terminus of the miRNA sequence and a partially complementary region of a coding transcript (mRNA) [28,29]. Nucleotides 2–8 of miRNAs are referred as the “miRNA seed regions” and are usually assumed to base-pair with seed-complementary regions located within the 3’UTR of mRNAs. Such miRNA-mRNA interactions either cause translation inhibition or induce the degradation of targeted mRNAs by recruiting specific cytoplasmic effectors [28,29]. Indeed, non-canonical seed regions have also been reported, such as “centered” miRNA seeds [30]. Moreover, it has been demonstrated that base-pairing beyond the seed region significantly contributes to stabilize miRNA-mRNA interactions and can determine the diversification of miRNA target repertoire among miRNAs of the same family [31,32].

Most miRNAs are pervasively expressed across human tissues, whereas others are significantly enriched only in one or few tissues since tissue-specific factors tightly control their expression. Additionally, it is known that several miRNAs are expressed in a developmental stage-dependent manner [26,33].

MiRNAs play critical roles in all aspects of cellular biology, including cell cycle [34], angiogenesis [35], brain development [36], and cognitive processes [37]. Accordingly, unbalances in miRNA expression or alterations in their primary sequence can determine the onset of various diseases, including tumors [38]. The first evidence of miRNAs’ involvement in the pathogenesis of human cancers came from studies on chronic lymphocytic leukemia (CLL). Here, it was shown that approximately 69% of CLL cases presented deletions at chromosome 13q14, which hosted genes coding for miR-15a and miR-16-1 [39]. Later, the authors demonstrated that these two miRNAs modulate the expression of the oncogene BCL2 by targeting its mRNA, hence inducing apoptosis [40]. These facts evidenced that miRNAs could act as oncogenes (oncomiRs) or tumor suppressor (tsmiRs) genes, depending on whether they target tumor suppressors or oncogenes, respectively [38].

## 3. ADAR Affinity for miRNA Editing Sites

MiRNAs can undergo A-to-I RNA editing, which alters their primary structure. Based on the current literature, the A-to-I editing phenomenon has been reported to occur in over 550 human miRNA transcripts, with the majority of edited sites having been detected at very low levels (<5%) [41].

The presence of inverted Alu sequences in the stems of miRNA precursors significantly increases ADAR affinity, often leading to higher editing frequencies (≥15%) [42]. Looking at triplets comprising the −1 upstream and +1 downstream neighbor nucleotides of the edited A, a correlation between specific three-nucleotide motifs and ADAR-editing affinity has been found. Precisely, ADAR1 has been shown to have a higher affinity for −1 upstream neighbors consisting of U = A > C > G. Instead, no particular preference for +1 downstream neighbor has been observed [43]. Similarly, ADAR2 affinity for −1 upstream neighbor nucleotides was reported as U ~ A > C = G. However, contrary to ADAR1, ADAR2 also showed a +1 neighbor preference, consisting of U = G > C = A [44]. Consequently, ADAR1 and ADAR2 target different editing sites, with only a partial overlap [44,45]. In general, however, the UAG triplet was found as the most favored among others [45,46]. Deeper insights into this issue come from a recent structural study, which demonstrated that human ADAR2′s nearest neighbor preference is actually determined by nucleotide-amino acid interactions rather than local duplex stability [47]. Concerning the 5′ nearest neighbor of edited A, the study showed that the presence of a G or C at position −1 instead of U causes a steric effect on the protein backbone at glycine 489, hence decreasing the deamination rate. Regarding the 3′ nearest neighbor of edited A, it was observed that G interacts with ADAR2′s serine 486 through an H-bond, facilitating the enzymatic deamination. Such an event did not occur when G was substituted with A, C, or U [47].

Another relevant cue concerning the affinity of ADARs for editing sites is relative to nucleotides opposite to the edited adenosine. In terms of frequency, cytosine (C) has been found to oppose the edited A three times more than expected by the natural occurrence of A::C mismatches in RNA duplex. Since inosine naturally base-pairs with cytosine according to the Watson–Crick rule, the presence of a C opposite to the edited A contributes to the stabilization of the duplex once the conversion from A to I has occurred [45,46,48]. These facts might suggest that A::C mismatches cause an increase in editing frequency. Additionally, both the −1 and the +1 neighbors of the UAG triplet are usually found to base-pair with respective opposed nucleotides according to the Watson–Crick rule, probably suggesting the importance of a local stable primary structure surrounding the A to be edited [46].

## 4. Effects of A-to-I miRNA Editing on miRNA Biogenesis and Function

### 4.1. A-to-I Editing in Pri- and Pre-miRNAs

**Figure 2:**
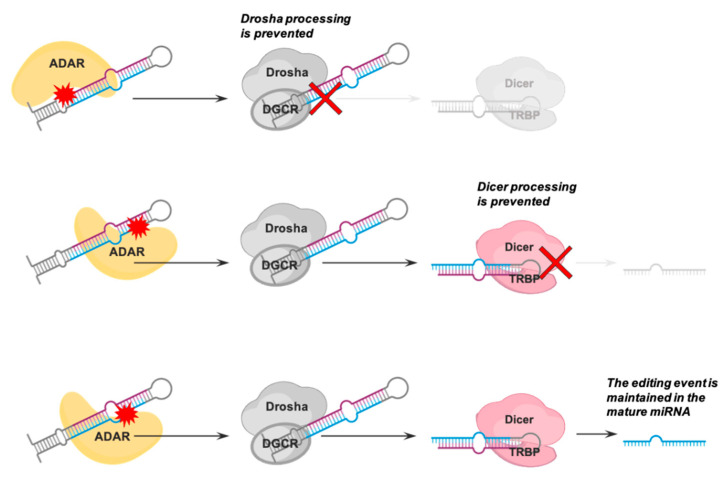
Steps of miRNA biogenesis at which adenosine to inosine (A-to-I) RNA editing can prevent the processing of pri- or pre-miRNA. In certain cases, however, the A-to-I editing event does not impair miRNA biogenesis and is maintained in the mature miRNA.

Editing of miRNA precursors can have significant implications on miRNA fate. Several studies reported that editing events in the pre-miRNA region could cause suppression or downregulation of miRNA biogenesis (Figure 2). Examples are editing events occurring at positions 11 and 14 of pri-let-7g, position 15 of pri-miR-33 [46], and positions 17 and 32 of pri-miR-455 [49] (positions are relative to the pre-miRNA sequence). This phenomenon is due to local structural conformation changes induced by editing across the miRNA precursor, preventing Drosha or Dicer from processing it. By performing an in vitro RNA editing assay, it was demonstrated that A-to-I editing of murine pri-miR-142 at positions 7 and 8 prevented the Drosha-DGCR8 cleavage, and thus the processing of this transcript [50]. Unprocessed edited pri-miR-142 transcripts were then rapidly degraded by the EndonucleaseV (EndoV)-Tudor-SN, a ribonuclease specific to inosine-containing double-stranded RNAs [50,51]. No interference in miRNA biogenesis was observed for A-to-I editing at positions 43 and 53 of the same precursor [50]. Deeper molecular insights were obtained in the case of human pri-miR-151a and its homolog in the mouse. Pri-miR-151a can be edited at positions 46 and 49. Although both these editing sites fall into the 3’ stem region, the one at position 46 may be more critical for the maintenance of the original secondary hairpin structure, as it is located at the border with the loop region and is paired with a uracil (A::U) [52]. By performing an in vitro dicing activity assay and a single-particle electron microscopy reconstruction, a recent study demonstrated that the editing site at position 46, but not that at position 49, is responsible for Dicer cleavage suppression [53]. In particular, it was shown that inhibition in dicing activity depended on a significant structural change occurring in the loop of pre-miR-151a edited at position 46. This caused a different conformational state in Dicer’s DExH/D domain, responsible for RNA duplexes unwinding, allowing their enzymatic cleavage. Instead, only two cases were reported in which editing of miRNA precursors caused an enhancement of the biogenesis process [46].

### 4.2. A-to-I Editing in Mature miRNAs

**Figure 3:**
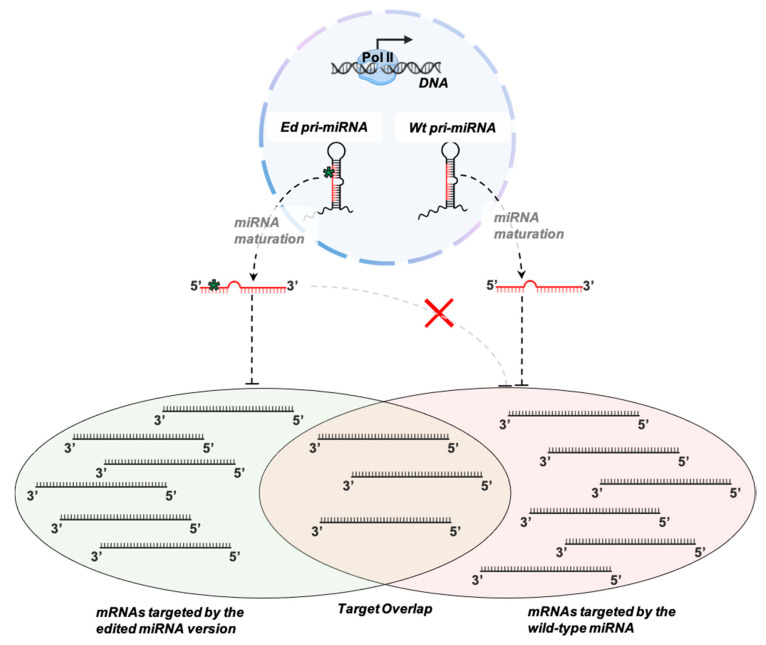
Schematic illustration of miRNA retargeting caused by editing of mature miRNAs. RNA editing can affect the miRNA-mediated modulation of gene expression by modifying the primary structure of wild-type (Wt) mature miRNAs. Such an event generates edited (Ed) miRNAs, often destroying their base-pairing with several complementary regions in the 3′UTRs of their original targets and creating complementarity for new targets. However, both the Wt and Ed miRNAs seem to share a subset of their target mRNAs.

A-to-I editing of pri-miRNAs can also lead to functional changes in mature miRNAs. Several editing events occurring within the mature sequence are maintained until the final stage of miRNA maturation, giving rise to edited mature miRNAs. Since miRNAs’ role in translational repression is conferred by sequence complementarity between the miRNA seed region and seed-complementary regions within the target mRNA, even a single nucleotide substitution along the mature sequence can determine changes in miRNA target repertoires, especially when editing sites fall into the seed region. This phenomenon is known as miRNA retargeting or target redirection, in which an editing event can create novel miRNA targets and/or destroy complementarity between a miRNA and the UTR of its canonical targets (Figure 3). Noteworthily, target redirecting seems not to cause a complete distinction in miRNA targeting between the wild-type and edited version of the same miRNA, but a target sharing of ~35% was estimated [54]. MiRNA retargeting in humans have been reported for miR-376a-5p edited at position 3 (redirecting from RAP2A to AMFR) [55], miR-200b-3p edited at position 5 (redirecting from ZEB1 to LIFR) [56], miR-589-3p (redirecting from PCDH9 to ADAM12) [57], and a few others. Finally, certain A-to-I editing events in miRNAs could cause simultaneous impairment of miRNA biogenesis and target redirecting, as might be the case of human miR-379-5p edited at position 5 [46,58].

In this context, a relevant limitation regards the expression level of edited mature miRNAs, which might create a bias in the study of edited miRNAs’ impact on human cancers. As a matter of fact, the vast majority of studies on miRNA editing have been focused on the editing level, a parameter obtained by normalizing the expression of the edited miRNA to the miRNA expression itself (i.e., the sum of both the wild-type and edited forms). Such a parameter is dependent on the expression of the wild-type form, hence carrying a bias that may compromise the final interpretation. To overcome this limitation, Nigita et al. [59] recently applied a new approach for miRNA editing measurement, which simultaneously considers the editing level and the absolute expression of the miRNA, assessed via reads per million (RPM) reads mapped to miRNAs. Noteworthily, the authors proved this method to be more efficient than the conventional method in distinguishing normal and tumor tissues in both types of lung cancer.

## 5. Detection of A-to-I miRNA Editing

### 5.1. General Issues

The analysis of miRNA transcripts for A-to-I editing detection requires the prior conversion of RNAs into complementary DNA (cDNA) molecules, followed by their amplification through polymerase chain reaction (PCR) to obtain small cDNA libraries. Such an aim is achieved through reverse transcription (RT), catalyzed by retroviral reverse transcriptases. During RT, the reverse transcriptase incorporates the cDNA nucleosides that address the Watson–Crick base-pairing rule [60]. When edited RNAs are retro-transcribed, the RT process incorporates nucleotides complementary to the modified nucleotide, causing an apparent misincorporation. In the case of A-to-I editing, a C is incorporated in the opposite strand of the cDNA in correspondence with the I. Subsequently, the first-strand of the cDNA will present an A-to-G change compared to the unedited RNA sequence, leaving a detectable trace of the RNA modification [61]. Once generated, cDNAs are sequenced to generate RNA reads, i.e., inferred sequences corresponding to part of a single RNA fragment. Finally, reads are mapped against an opportune genomic DNA to identify real RNA modifications, distinguishing them from genomic mutations [61].

Although the procedure of miRNA editing detection might seem relatively straightforward, it presents several challenges. These are essentially represented by the necessity of minimizing both technical (e.g., chimeras, sequencing errors, read misalignments) and biological (e.g., genomic polymorphisms, rare somatic mutations) biases, which might result in the erroneous estimation of the editing activity. The accurate identification of miRNA editing sites hence this requires ad hoc, adjustable computational methodologies.

A decrease in sequencing errors is undoubtedly obtained by applying stringent filters during preprocessing steps and variant calling, retaining only high-quality reads and mismatch events supported by multiple reads [62]. More effective results can be achieved by increasing coverage and sequencing accuracy, which leads to higher sensitivity and more robust statistical support [63]. In this context, worthy of note is the differential expression of miRNA transcripts and biases introduced during cDNA amplification (PCR) or sequencing, as these parameters affect the uniformity of read coverage [64,65]. Here, the availability of biological and technical replicates is useful to minimize or even solve such drawbacks [64].

The reliability of reads alignment is fundamental for the identification of miRNA editing. At the beginning of the application of high-throughput sequencing (HTS) technologies in the field of miRNA editing detection, the cross-mapping of edited miRNA reads represented a major issue [66]. Cross-mapping consists of the accidental alignment of edited RNA sequences originating from one locus to a different one. Due to their limited length, cross-mapping tends to be recurrent in edited miRNA reads, especially in the case of miRNAs derived from repeat RNAs or belonging to the same family. However, several adjustments have been introduced in modern bioinformatic pipelines that overcome such a snag (e.g., [66,67]). Additionally, the use of opportune sequencing strategies is influential in the mapping of edited miRNA reads. For example, using technologies that allow the generation of longer reads—in the case of pri- or pre-miRNA transcripts—and replacing single-end with paired-end sequencing mode increases the accuracy of editing detection [68,69]. In particular, combining the paired-end sequencing of miRNAs with RNA immunoprecipitation sequencing (RIP-seq) of A-to-I RNA editing enzymes greatly improves the sequencing resolution [69].

Different biases might also affect the variant calling. RNA editing is identified based on single mismatches revealed during the alignment between RNA reads and the reference genome. However, such an occurrence might represent the result of a single-nucleotide polymorphism, a somatic mutation, or a sequencing artifact rather than a real editing event [62,63]. From this standpoint, some strategies have been adopted to reduce the probability of false-positive miRNA editing calls. For instance, reliable identification of miRNA editing sites can be obtained by mapping miRNA reads against DNA sequenced from the same individual [62]. Here, the combination of pre-aligned RNA-Seq and matched DNA-Seq reads allows reaching a high accuracy level [70].

On the other hand, the ever-increasing number of available high coverage multi-sample data sets enables identifying and removing most genomic variations, even in the absence of DNA-seq data. For example, a mismatch that recurs in a miRNA across multiple samples is unlikely to result from genomic mutations [71]. Moreover, in the case of miRNA reads that harbor pairs of single-nucleotide variants, an analysis of allelic linkage between single-nucleotide variants can be used to distinguish genomic polymorphisms from real miRNA editing events [72].

### 5.2. High-Throughput Methods with Higher Accuracy

Decreases in sequencing errors can also be reached by employing the recently developed Inosine Chemical Erasing sequencing (ICE-seq) method, which showed higher accuracy than conventional HTS [73,74]. This method is based on the inosine-specific cyanoethylation by acrylonitrile, which causes RT’s stoppage, allowing genuine A-to-I conversions to be distinguished from sequencing artifacts. Although, at present, this technique has only been exploited for the identification of A-to-I sites in poly-A+ transcripts, its protocol can be adapted for poly-A- RNAs, including miRNA transcripts [75]. Editing frequency and neighboring editing sites are the major limiting factors for this method [75]. Similarly, in the recently developed EndoV inosine precipitation enrichment sequencing (EndoVIPER-seq) method, a recombinant version of the Escherichia coli EndoV (eEndoV) is used to isolate A-to-I edited RNAs through adjusted cationic conditions [76]. Here, the presence of relatively high concentrations of calcium ions (Ca2+) causes the binding of eEndoV to inosine substrates instead of eEndoV-mediated catalytic cleavage. EndoV-bound edited RNAs are then precipitated by magnetic immunoprecipitation and finally sequenced by HTS [77].

## 6. Current Ad Hoc Bioinformatics Methods for A-to-I miRNA Editing Detection

At the early stage of the study on A-to-I miRNA editing, Sanger sequencing represented the conventional method allowing a reliable identification and quantification of miRNA editing [78]. However, though ensuring high accuracy, this technique is often time-expensive and enables the sequencing of a restricted set of miRNA molecules [46], even when using automated, paralleled capillary gel electrophoresis systems [79]. As such, Sanger sequencing would be more suitable for targeted approaches and is currently being used as a validation and quantification method for known or putative A-to-I miRNA editing sites [42,78].

Unlike Sanger sequencing, HTS technologies allow the time-effective processing of massive small RNA libraries, with a drastic improvement in the detection and quantification of editing events and miRNA isoforms. On the other hand, HTS technologies present a higher risk of false-positives and strictly depend on optimized bioinformatics pipelines to correctly process RNA reads [80].

At first, bioinformatics procedures for miRNA editing identification were prone to several biases as they did not include appropriate corrections and adjusted parameters (e.g., [81,82]). In this context, de Hoon et al. developed the first pipeline corrected for cross-mapping, allowing a much reliable detection of editing events in mature miRNA sequences [66]. A remarkable refinement of such a bioinformatic approach was then obtained by Alon et al. [67], specifically focused on A-to-I instances. Here, the trimmed miRNA reads are mapped against the reference genome using the Bowtie tool [83], retaining unique best hits with up to one mismatch and reads aligned to genomic loci of known miRNAs. Then, a stringent filter discards reads with low-quality scored mismatches, and finally, the sequencing error rate is estimated [84].

More recently, Zheng et al. developed MiRME [85], a valuable, comprehensive method for detecting miRNA mutations and editing sites. Besides using the cross-mapping correction method, the MiRME workflow is based on three progressive sequence alignment rounds. This strategy allows for reaching high sensitivity though maintaining a low computational time. MiMRE permits the identification and visualization of all types of putative editing types and single nucleotide mutations. Similarly, Lu et al. implemented miRge 2.0 [86], a method dedicated to detecting and quantifying miRNAs, A-to-I miRNA editing events, and isomiRs (i.e., miRNA isoforms). Here, novel miRNA detection is based on a machine-learning algorithm that increases specificity. After performing reads, annotation, and mapping, the mapped output file undergoes A-to-I editing analysis. During this phase, potentially edited miRNA sequences are discarded if they cross-map, their canonical sequences are expressed at low levels (<1 RPM), or they can be aligned to more than one location in the genome by trimming the last two nucleotides at the 3′ end.

## 7. Validation and Functional Characterization of miRNA Editing

### 7.1. Available Validation Methods

Using validation techniques to verify the variant calling of selected editing sites is useful for reducing the risk of false positives. To date, several validation methods exist, each based on a different biochemical/biological principle. They include Sanger sequencing of RT-PCR products, single-base primer extension (SNaPshot) assay, inosine (I)-specific cleavage of RNA, and ADAR perturbation experiments (Table 1). As a general rule, the more the validation method differs in its principle from the initial detection method, the greater the confidence of validation will be. However, each validation method presents limited sensitivity, requires increased amounts of work, and is usually time-expensive, limiting the number of sites that can be reasonably validated. Additionally, little information is available regarding the specific advantages and disadvantages of techniques used to validate candidate editing sites from transcriptome-wide analyses. Hence, no optimal solution is known at present.

Among the targeted approaches, Sanger sequencing of edited miRNA transcripts (pri-miRNAs, pre-miRNAs, or mature miRNAs) currently represents the most used validation method among the conventional ones. pri-miRNAs are sufficiently long to undergo standard reverse transcription (RT) and the subsequent PCR amplification of cDNAs containing the editing sites followed by the subcloning of PCR products and sequencing [78]. By contrast, pre-miRNAs and mature miRNAs necessitate the addition of a poly(A) tail at the 3‘ end and the ligation of a 5‘ adaptor RNA due to their sequences’ brevity. The RT is performed using an oligo(dT)30 bound to a linker sequence at 5‘ end. The derived cDNAs are then amplified, employing specific internal primers complementary to pre-miRNA or mature miRNA sequences. Finally, the PCR products are subcloned and subject to sequencing [78].

SNaPshot assay [87] constitutes another valuable targeted approach for editing site validation. This method is a sensitive tool that potentially allows the validation of over 30 putative modification sites scattered in multiple miRNA molecules in a single reaction [88]. Here, ad hoc primers are designed to hybridize to target complementary regions of the nucleic acids. Each primer is designed so that its last base at the 3’ end position itself adjacent to the editing site to be validated. Using a DNA polymerase solution and the four (A, G, T, and C) labeled dideoxynucleotides (ddNTPs), the primers are enzymatically extended by only one base. The labeled ddNTPs are incorporated at the 3’ end of the primers according to the Watson-Crick complementary rule and then examined to validate the variant calling. The SNaPshot technique was recently exploited to validate a set of hypo-edited A-to-I miRNA editing sites [89].

I-specific cleavage aims to obtain the enzymatic cut of edited RNAs in the inosine correspondence, with no possibility of off-target. The first developed I-specific cleavage strategy envisages glyoxal reaction with inosines and guanosines along the RNA molecule. Here, the G adducts are chemically stabilized by high borate concentrations in the reaction solution, contrary to the I adducts. Finally, the protocol envisages the cleavage of RNA molecules at the level of glyoxalated I and the RT-PCR and sequencing of RNA fragments [90]. Later, the use of Endonuclease V (EndoV) was repurposed to carry out validation of A-to-I editing sites by I-specific cleavage [91].

An indirect validation of putative editing sites can be achieved by comparing signals generated in non-manipulated organisms (cell lines or mice) versus corresponding signals in ADAR-depleted organisms (e.g., [46,57,67]). This method offers the potential to validate a broad set of candidate A-to-I sites, albeit it is generally considered a weak validation method which only permits indirect confirmations. Noteworthily, knockout experiments are by far preferable to RNA interference-based knockdown since the latter leaves an uncertain residual level of editing activity, hence reducing accuracy. Indeed, the application of this technique in vivo presents a notable drawback, as the double knockout of ADAR in mice embryos can result in lethality. Such a limitation can be overcome by using tamoxifen-inducible transgenic mice (e.g., [92]).

### 7.2. Approaches for Functional Characterization

As previously stated, A-to-I editing might cause the stoppage of miRNA biogenesis or a shift in miRNA-targeting if it occurs within the mature sequence, especially within the seed region. To investigate the biological role of an A-to-I event, researchers can proceed by carrying out ascertained methods allowing the functional characterization of such an instance.

An in vitro assay can be performed to investigate the role of A-to-I editing in miRNA biogenesis (e.g., [50,52]). After an A-to-I site has been identified and validated, a plasmid containing the pri-miRNA sequence is added to an editing reaction mixture containing ADAR and used for in vitro transcription. After incubation, the in vitro edited primary miRNA is first subjected to a Drosha–DGCR processing and later to a Dicer–TRBP reaction. The reaction products are finally examined by cloning and sequencing the cDNA isolates. As an alternative, edited and unedited versions of the primary miRNA can be synthesized in vitro, labeled with radioisotopes or dyes at appropriately chosen sites, and then subjected to the Drosha-DGCR8 and Dicer-TRBP processing. The final products are finally examined by electrophoresis and compared to each other [50,52].

The standard procedure to assess the functional consequence of editing events within mature miRNAs [93] requires a robust miRNA-target binding prediction analysis for both the miRNA versions (unedited and edited). Currently, several algorithms exist that are capable of inferring putative miRNA targets. These can be based on sequence complementarity, thermodynamic stability, evolutionary conservation, statistical inference, or their combination [94]. Once the list of target genes for the edited and unedited miRNAs has been generated, a Gene Ontology (GO) enrichment analysis is performed to infer the potential impact of single or multiple miRNA editing events on specific biological pathways [93]. To facilitate this process, our group has lately developed isoTar, a Web-based containerized application designed for performing consensus miRNA targeting prediction and functional enrichment analyses [95]. isoTar allows users to exploit the potentialities of five commonly used tools for miRNA targeting prediction with customized statistical thresholds. The list of putative target genes can be downloaded or further processed for a functional enrichment analysis with a few clicks. At the end of the predictive computational process, the putative target genes with higher consensus/scores are evaluated by custom miRNA mimics transfection experiments through methods that determine their expression levels [93]. In particular, quantitative RT-PCR of custom edited miRNAs is used to test the fluctuation of candidate target mRNAs [96], while western blotting is used to analyze and compare protein expression levels of putative target genes in case versus control [97]. Alternatively, the validation of mRNA repression by edited miRNA can be performed through a luciferase reporter assay [98], optionally followed by an experiment of binding region mutagenesis [99].

## 8. Relevance of A-to-I miRNA Editing to Human Cancer

**Figure 4:**
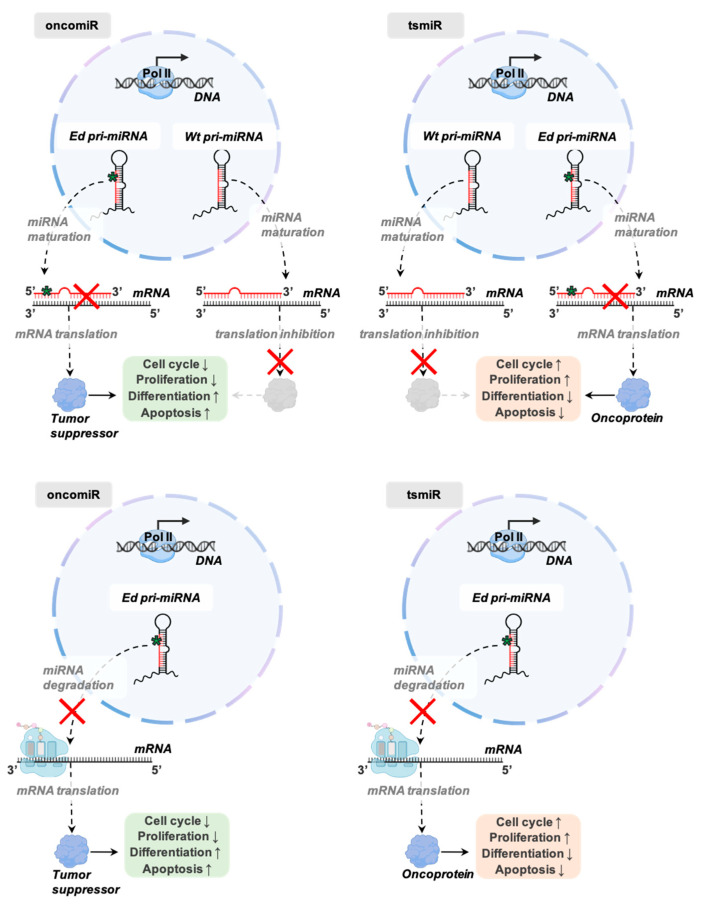
Possible effects of miRNA editing in the context of cancer. Editing of mature miRNAs can prevent the targeting of complementary regions in the 3′UTRs of original mRNAs. In case an editing event disrupts the base pairing of an oncomiR with its target mRNA (transcribed from a tumor suppressor gene), the translational process will lead to the expression of the tumor suppressor protein, increasing processes related to cell cycle arrest, differentiation, and/or apoptosis (top, left). In case an editing event disrupts the base pairing of a tsmiR with its target mRNA (transcribed from a proto-oncogene), the translational process will lead to the expression of the oncoprotein, increasing processes related to cell cycle progression, proliferation, and migration (top, right). However, editing of pri- or pre-miRNAs can cause the blockage of their biogenesis, significantly decreasing the expression of their related mature miRNAs. Thus, similar considerations can be done in the case of the underexpressed miRNA functions as an oncomiR (bottom, left) or a tsmiR (bottom, right).

As a modulator of miRNA expression and function, A-to-I miRNA editing exerts a valuable impact in reference to the oncological field (Figure 4) and has been suggested as a potential biomarker for cancer prognosis and therapy [54,56]. For example, it was recently reported that miRNA editing in cancer tissues is downregulated while editing of mRNAs’ 3′ UTR is upregulated. In the same study, the authors showed that elevated miRNA editing levels are associated with longer survival [54]. However, the precise biological meaning of miRNA editing depends on the specific context in which it occurs. For instance, it was demonstrated that under physiological conditions, pre-miR-455 is highly edited at positions 17 and 32 in human melanocytes [49]. This causes the blockage of its biogenesis and the subsequent degradation of the edited precursors, leading to negligible levels of unedited miR-455-5p. Instead, during melanoma progression, A-to-I editing of pre-miR-455 dramatically decreases due to the CREB-mediated inhibition of ADAR1 expression [100,101]. This leads to a significant increase of unedited miR-455-5p, which specifically targets the CPEB1 binding protein, a notorious regulator of translation that functions as a tumor suppressor gene, thus enhancing melanoma growth [49].

Differently from the case of miR-445, A-to-I editing of pre-miR-200b at position 61 (corresponding to position 5 in miR-200b-3p) was demonstrated to correlate with poor prognosis in several classes of cancer patients, as it promotes processes related to cell migration and invasiveness in various tumor types [56]. Under physiological conditions, miR-200b-3p is mainly found in its unedited form, functioning as a suppressor of genes related to metastatic processes. These include ZEB1 and ZEB2, two transcription factors that lead to the expression of master regulators of epithelial-mesenchymal transition [102,103]. On the contrary, under oncological conditions, A-to-I editing of pre-miR-200b at position 61 significantly increases, leading to the expression of edited miR-200b-3p. Such an occurrence causes a change in the functional role of miR-200b-3p, which loses the ability to target several oncogenes, including ZEB1 and ZEB2, while gaining the ability to target a new set of coding transcripts, including LIFR, which acts as a tumor suppressor in several circumstances [56]. An opposite case is that of miR-589-3p [57]. Pre-miR-589 is almost entirely edited at position 66 (position 6 in miR-589-3p) in the normal brain tissue, and its mature form functions as an oncosuppressor gene by inhibiting the expression of ADAM12. This metalloproteinase promotes cell proliferation, mobility, and invasion in various cancer types, including brain cancer. Differently, in glioblastoma tissues, editing of miR-589-3p undergoes a consistent decrease due to the underexpression of ADAR2, leading to higher expression levels of wild-type miR-589-3p. The latter lose the ability to target ADAM12 while gaining the ability to target PCDH9, a tumor suppressor associated with glioma progression, boosting brain cancer cell proliferation and invasiveness [57].

In some circumstances, target redirecting could relate to the targeting of distinct regions of the same 3’UTR in an mRNA. This is the case of unedited miR-27a-3p and its edited version at position 5 [104]. By employing a luciferase assay, it was demonstrated that both these versions of miR-27a-3p were capable of targeting the MET transcript, apparently with the same efficacy, albeit at two distinct miRNA-targeting sites. However, edited miR-27a-3p also displayed a functional shift, as it lost the ability to target the EGFR transcript and contributed to phenotype alteration in MCF-7 cells [104]. 

Besides redirecting events, ADAR-mediated impairment of miRNA biogenesis also contributes to cancer biology. For example, editing of mir-26a-1 at position 74 drives the proliferation of normal human hematopoietic progenitors by impairing the miRNA maturation [105]. miR-26a-3p directly targets the EZH2 transcript. EZH2 participates in histone methylation. Its expression is tightly associated with cell proliferation [106] as it suppresses the expression of genes related to cell cycle control, including CDKN1A [105]. Conversely, enforced miR-26a-3p expression significantly prevented the self-renewal capability of chronic myeloid leukemia, confirming a role for miR-26a-3p as a relevant tumor suppressor [105]. Differently from the case of mir-26a-1, the ADAR2-mediated editing of mir-222 (positions 10 and 83) and mir-221 (positions 24, 25, and 88) precursors, two notorious oncomiRs, inhibit their maturation and are essential for the maintenance of physiological conditions [107]. During glioblastoma progression, ADAR2 expression is severely decreased, implying significantly lower editing levels in tumor cells. The ADAR2 dysregulation leads to the subsequent maturation of miR-222-3p and miR-221-5p, which correlates with increased proliferation and migration of glioblastoma cells. Conversely, the rescue of ADAR2 activity in glioblastoma cells rebalances the expression of several miRNAs, including miR-222-3p and miR-221-5p, restoring the expression levels observed in normal human brain cells [107].

Aside from these mentioned case reports, only a few other studies have demonstrated the importance of miRNA editing at the pathophysiological level [41]. Indeed, the vast majority (>99%) of miRNA editing events reported until nowadays in both humans and mice have remained functionally uncharacterized, despite their potential in determining the shift between physiological and pathological status [41]. Moreover, it still remains to elucidate the impact of miRNA expression on the physiological consequences of miRNA editing. For instance, a given editing site falling within a miRNA seed region might show an elevated editing level (>15%), though the absolute expression of the miRNA could be very low (<1 RPM). This would likely cause only a minimal impact on the miRNA targetome [59]. Overall, these facts highlight the need to employ effective approaches capable of detecting editing and determining the real biological meaning of single editing events across miRNAs.

## 9. Discussion

Over the last fifteen years, miRNA editing has attracted an ever-increasing number of researchers in the biomedical field. A-to-I miRNA editing is a finely regulated mechanism that influences cellular physiology and is relevant to cancer biology. Both ADAR1 and ADAR2 have been recently identified as interesting oncotargets due to their involvement in oncogenic processes, either by editing of coding or non-coding transcripts like miRNAs. In line with these statements, several studies have shown that differential miRNA editing has a potential to be exploited as a predictive and prognostic biomarker of disease, including cancer. Nonetheless, there are still limitations and open questions in the field, as briefly discussed hereafter.

To date, a large number of A-to-I miRNA editing events have been reported in the literature, though most of them have not been validated yet, and the vast majority of such instances have remained functionally uncharacterized, which is a considerable limitation. Putative miRNA editing sites might represent technical artifacts rather than genuine editing events, outweighing the editing signal. This challenges the correct identification and contextualization of miRNA-editing patterns in human cancers. Accurate validation of putative editing sites certainly represents the lynchpin to this issue. Nonetheless, at present, only methodologies such as Sanger sequencing, SNaPshot, and targeted I-specific cleavage have been applied to meet such need. As previously anticipated, each of these techniques has its own drawbacks, making them disadvantageous when validation has to be carried out for a wide set of putative editing sites, as is the case for small RNA-seq variant calls. Recently developed methodologies like RIP-seq, ICE-seq, and EndoVIPER-seq might represent a satisfactory compromise between number of detected editing sites and detection reliability. On the other hand, however, these methods are more expensive, require skilled labor, and add a further layer of complexity to the experimental setting. Additionally, the application of these sequencing technologies to miRNA editing might require further adaptation. In addition to the mentioned methods, Nanopore sequencing technologies could allow the direct detection of inosines in full-length pri-miRNAs, pre-miRNAs, and intronic miRNAs, overcoming several technical issues that hamper editing detection, including those linked to PCR amplification [108,109].

The functional characterization of edited miRNAs has been widely overlooked, despite being the core issue of this research field. To date, only the 0.8% of miRNA editing evets have been experimentally characterized. In this context, structural studies exploring how A-to-I editing can alter the spatial conformation of pri- and pre-miRNA duplex at various instances might reveal the basis for accurately predicting whether a site-specific editing event would result in a decrease, increase, or even suppression of miRNA biogenesis.

Similar studies might investigate the role of ADAR1-mediated editing of pri- and pre-miRNAs in the context of cytosolic innate immune response, which is relevant to cancer biology. Precisely, tumor cells promote the infiltration of interferon-producing immune cells and often become themselves a source of interferon production [10,110]. Interferons are pro-inflammatory cytokines that stimulates the expression of other pro-inflammatory agents, such as interleukins and tumor necrosis factors. The systemic effects of pro-inflammatory cytokines are associated with tumor progression, fatigue, chemotherapy resistance, and cancer cachexia in advanced tumor patients [110,111]. In recent years, there has been evidence that in certain circumstances miRNAs can be recognized by notorious innate immune RNA sensors like RIG-I/MDA5 and Toll-like receptors (TLRs) [112,113,114,115], leading tumor cells towards an interferon-stimulated gene expression pattern. In this context, ADAR1-mediated editing could exert a regulatory role, ideally preventing possible autoimmune responses linked to miRNA-immune RNA sensors interactions. Indeed, such occurrences are largely unexplored, and certainly deserve to be explored.

Finally, the development of accurate bioinformatics pipelines and resources for miRNA editing, such as robust prediction tools assessing the potential functional impact of edited miRNAs, are of great support for researchers in the field. In particular, the construction of a dedicated comprehensive online repository for miRNA editing could increase the reliability of various putative editing sites by the consensus of a high number of site-specific editing signals from different studies. Additionally, such a data repository would represent a starting point for future experimental setting. The development of such bioinformatics resources, thus, should be encouraged.

## 10. Conclusions

Disease-associated RNA recoding sites are anticipated to become soon a major target of the biomedical research, especially in cancer, autoimmune diseases, and viral infections. In the present article, we briefly discussed approaches and methods for miRNA editing detection, validation, and characterization to improve our understanding of the potential role of A-to-I edited miRNAs in human cancers. Edited miRNAs might represent efficient diagnostic and prognostic biomarkers of cancer development and progression, lending itself to be used as therapeutic targets. Although current HTS technologies allow single-nucleotide resolution and precise mapping of editing sites, there is still a need to develop more effective methodologies and strategies to identify, validate, and analyze better editing events in miRNAs. In the future, precision medicine based on epitranscriptome signatures such as miRNA editing may be tailored to specific tumor types. Hence, more efforts should be made in the coming years to characterize miRNA editing in normal and cancer conditions, providing the opportunity to develop more sophisticated diagnostic and prognostic procedures in the clinical practice.

## Figures and Tables

**Table 1:** Major characteristics of validation methods listed in this chapter.

Validation Method	Biochemical/Biological Principle	Main Features
Sanger sequencing	DNA synthesis reaction using a mixture containing the four dNTPs and chain terminating labelled ddNTPs in established concentrations.	Targeted approach; direct; need of customized optimization; time-consuming; applicable to multiple editing types.
SNaPshot	Extension of primers complementary to selected cDNAs by one base (in correspondence of the modified base) in a reaction solution containing the four dNTPs and labelled ddNTPs.	Targeted approach; direct; no need of customized optimization; time-effective; applicable to multiple editing types.
I-specific cleavage (chemical-enzymatic approach)	Glyoxalation of guanines and inosines and subsequent cleavage of inosine adducts by Ribonuclease T1. Guanosine adducts are protected by borate.	Targeted approach; direct; no need of customized optimization; time-effective; specific for A-to-I.
I-specific cleavage (enzymatic approach)	Cleavage of A-to-I edited RNAs by EndoV, the ribonuclease specific to inosine-containing RNAs.	Targeted approach; direct; no need of customized optimization; time-effective; specific for A-to-I.
ADAR KD	Downregulation of ADAR expression by RNA interference (RNAi) mechanism.	Wide-range approach; indirect; need of customized optimization; time-consuming; specific for A-to-I.
ADAR KO	Total suppression of ADAR expression by gene loss or inactivation.	Wide-range approach; indirect; need of customized optimization; time-consuming; specific for A-to-I.
